# Comparison of the microbial population in rabbits and guinea pigs by next generation sequencing

**DOI:** 10.1371/journal.pone.0165779

**Published:** 2017-02-09

**Authors:** Edward J. Crowley, Jonathan M. King, Toby Wilkinson, Hilary J. Worgan, Kathryn M. Huson, Michael T. Rose, Neil R. McEwan

**Affiliations:** 1 Institute of Biological, Environmental and Rural Sciences, Penglais Campus, Aberystwyth University, Aberystwyth, Wales; 2 Ystwyth Veterinary Group, Llanbadarn Fawr, Aberystwyth, Wales; Central South University, The Third Xiang Ya Hospital, CHINA

## Abstract

This study aimed to determine the microbial composition of faeces from two groups of caecotrophagic animals; rabbits and guinea pigs. In addition the study aimed to determine the community present in the different organs in the rabbit. DNA was extracted from seven of the organs in wild rabbits (n = 5) and from faecal samples from domesticated rabbits (n = 6) and guinea pigs (n = 6). Partial regions of the small ribosomal sub-unit were amplified by PCR and then the sequences present in each sample were determined by next generation sequencing. Differences were detected between samples from rabbit and guinea pig faeces, suggesting that there is not a microbial community common to caecotrophagic animals. Differences were also detected in the different regions of the rabbits’ digestive tracts. As with previous work, many of the organisms detected were Firmicutes or unclassified species and there was a lack of Fibrobacteres, but for the first time we observed a high number of Bacteroidetes in rabbit samples. This work re-iterates high levels of Firmicutes and unclassified species are present in the rabbit gut, together with low number of Fibrobacteres. This suggests that in the rabbit gut, organisms other than the Fibrobacteres must be responsible for fibre digestion. However observation of high numbers of Bacteroidetes suggests that this phylum may indeed have a role to play in digestion in the rabbit gut.

## Introduction

The digestive tract of all animals contains a diverse range of microbes, many of which share a symbiotic relationship with their hosts. For example the presence of gut microbes is considered essential in increasing the host animal’s access to nutrients within their feed. In turn by ingesting food, the host animal provides the microbes with a constant supply of nutrients and a homeostatic environment. Although the host-microbe relationship is important in the digestive tract of all animals, probably many of the best studied examples of this symbiotic relationship can be seen in herbivores which have a high dependency on fibrous food such as grass.

It is long-established that the digestive tract of the rabbit has a bacterial community, and more recently it has also been shown that there is an archaeal population [1]. In many herbivorous species there are also eukaryotic microbes within the gut population; both ciliated protozoa and fungi. Both of these populations have been described in the hindgut of horses and foregut of ruminants and are known to be monophyletic [2], [3], but neither eukaryotic population has ever been described in the digestive tract of the rabbit.

Within the herbivorous species there are differences in their digestive anatomy and physiology. In general, these herbivores can be sub-divided into three general categories: foregut digesters such as ruminants and camelids; hindgut digesters such as equids and rodents; and caecotrophagic animals such as rabbits and hares. These animals show differences in the length of time taken to digest food, with the rabbit having the shortest mean retention time, and ruminants having the longest [4]. Given the anatomical and physiological differences between the groups of animals, it was not surprising that the bacterial community of the equine hindgut was different from that of the rumen [5], and likewise the bacterial community of the rabbit hindgut was different from that seen in either the horse’s hindgut or the ruminant’s rumen [6].

The increasing accessibility of next generation sequencing (NGS) has meant that the microbial diversity of more and more ecosystems can be examined and the organisms within them identified. Here we present a comparison between the bacterial community of faecal samples from the rabbit and the community of another caecotrophagic species, the guinea pig, to compare samples collected from different species of domesticated caecotrophagic animals, as well as samples collected from different regions of the digestive tracts of rabbits living in the wild.

## Results

In total 450,544 high quality sequences were obtained from the 12 faecal samples (6 rabbits and 6 guinea pigs) which clustered into 811 unique operational taxonomic units (OTUs); 396 were only found in rabbits, 301 were only found in guinea pigs, and 114 were found in samples from both species. In the case of the 35 samples from the wild rabbits (5 animals and 7 organs), 1,110,653 high quality sequences were obtained which clustered into 914 unique OTUs. Numbers of OTUs per phylum for each type of sample (organ or host species) are shown in Table 1. All sequence information has been deposited with the European Nucleotide Archive, with the accession numbers ERS1167047-ERS1167093. Information relating to sub-phylum distribution is contained in Table A in S1 File.

**Table 1:** Total number of OTUs detected in each sample group.

	Actinobacteria	Bacteroidetes	Chloroplast	Fibrobacteres	Firmicutes	Proteobacteria	Tenericutes	Unclassified
Guinea Pig Faeces (n = 6)	2	183	1	1	104	7	1	116
Domesticated Rabbit Faeces (n = 6)	10	67	2	0	239	8	0	184
Wild Rabbit Rectum (= 5)	12	98	2	0	378	10	1	284
Wild Rabbit Stomach (= 5)	7	113	2	1	245	9	0	154
Wild Rabbit Jejunum (= 5)	12	63	2	0	313	9	1	209
Wild Rabbit Appendix (= 5)	12	54	2	0	365	9	1	258
Wild Rabbit Caecum (= 5)	12	58	2	0	363	8	1	266
Wild Rabbit Proximal Colon (= 5)	12	38	2	0	355	9	0	254
Wild Rabbit Distal Colon (= 5)	12	43	2	0	355	8	1	252

### Comparison of domesticated rabbit and guinea pig faecal samples

**Fig 1:**
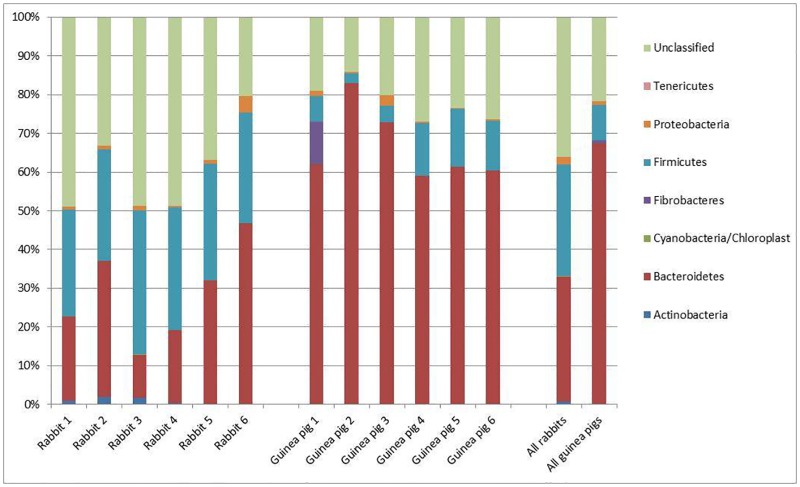
Percentage of each phylum present in fresh faecal samples collected from domesticated rabbits and guinea pigs together with the percentage of sequences which could not be classified within a particular phylum.

In addition to a number of chloroplast and unidentified sequences, six different phyla of bacteria were identified within the sequences: Actinobacteria; Bacteroidetes; Fibrobacteres; Firmicutes; Proteobacteria; and Tenericutes. Fig 1 shows the relative abundance of each of these phyla within the animals. Of these phyla most OTUs were identified within one of three categories: Bacteroidetes (32.3% in rabbits and 67.3% in guinea pigs); Firmicutes (29.0% in rabbits and 9.3% in guinea pigs); and unclassified bacteria (36.2% in rabbits and 21.6% in guinea pigs).

In total 203 OTUs were classified as being from the phylum Bacteroidetes. In addition to the density of Bacteroidetes in guinea pigs being approximately double that of the rabbits, the number of OTUs specific to guinea pigs (136 OTUs) was higher than the number of rabbit-specific Bacteroidetes (20 OTUs) or those found in both species (47 OTUs). Although there are 47 OTUs which are present in faecal samples from both species, it is worth noting that in all cases the OTU is seen as more abundant in one species than the other (typically 100–1000 times higher in the preferred species). Thus, although the Bacteroidetes are present in samples from both species, there seems to be a sub-set within this phylum which is specific to, or at least over-represented in, either the guinea pig or rabbit.

The other major phylum detected comprised the Firmicutes. These sequences could be categorised as 312 distinct OTUs; 208 of these were only seen in rabbits, 73 only in guinea pigs and 31 were observed in both species. As with the Bacteroidetes, the Firmicutes OTUs that were found in samples from both species showed a higher abundance in one species than the other (again typically 100–1000 times higher in the preferred species). Therefore, although the Firmicutes are also present in samples from both species, there again seems to be a sub-set within this phylum which is specific to, or at least over-represented in, one or other species.

No Fibrobacteres were observed in any of the faecal samples from rabbits. However, a single Fibrobacteres OTU was seen in four of the guinea pig samples, albeit at only a trace level (single copies) in three of them. In the samples from the remaining guinea pig, the Fibrobacteres sequences constituted approximately 11% of the entire population.

Proteobacteria were seen in all twelve samples analysed. In no animal did the abundance of the Proteobacteria exceed 4.3%. In total 13 proteobacterial OTUs were observed, with 6 found only in rabbits, 5 only in guinea pigs, and 2 in both. However, unlike the Bacteroidetes and Firmicutes, there was little evidence of quantitative segregation of shared OTUs within this phylum. Instead each animal had a few proteobacterial OTUs, often with one OTU being more abundant than others, although the OTU which was most abundant varied between animals, irrespective of species.

Actinobacteria were found in faecal samples from both species, and although they were at low abundance in both species, they were more abundant in rabbits (0.8%) than in guinea pigs (0.06%). In total, 11 different actinobacterial sequences were observed. One of these (OTU0237) was found in samples from both species, and was found in all rabbits (around 85% of the total actinobacterial sequences in rabbits), although it was only found as a single sequence in one of the guinea pig samples. In addition, it was found as the only actinobacterial sequence in one of the rabbits. The other ten actinobacterial sequences were species-specific. OTU0641 was specific to the guinea pigs, and was found in the five animals which lacked OTU0237, and although present at low levels (0.2%) in two animals, in the remaining three it was only present at trace levels (1 or 2 copies). The remaining nine actinobacterial OTUs were restricted to the rabbits and were found at very low levels.

A single Tenericutes sequence (*Mycoplasma* sp.) was detected in one of the guinea pig samples, but none was detected in any of the faecal samples from rabbits.

In the case of the rabbit samples, the largest group of sequences was actually those which could not be classified at a phylum level (based on a minimum of 90% identity). The mean figure for all rabbit sequences showed that 36.2% of the sequences could not be classified, and this figure approached 50% of all sequences in three of the animals. The number of unclassifiable sequences in samples from guinea pigs was considerably fewer (21.8%) but was still the second largest group of organisms. In total 268 different unclassified OTUs were identified, with 152 of them being only detected in samples from rabbits, 84 only being identified in guinea pigs and 32 being found in samples from both species. As with the Bacteroidetes and Firmicutes, the OTUs identified in faecal samples from both species tended to show bias in favour of one species or the other (100 to 1000 fold difference), with the exception of two OTUs, one of which showed 100% identity to sequences derived from faecal samples from a number of mammalian species.

Only two OTUs corresponding to chloroplast sequences were seen. One derived from *Lolium multiflorum* (Italian ryegrass) was detected in five rabbit samples and five guinea pig samples. In addition, *Trifolium strictum* (clover) sequences were detected in five of the rabbit samples, but were absent from guinea pig samples. However, in all cases the chloroplast sequences never exceeded more than 0.13% of the total number for that particular animal.

Cochran’s Q test was used to provide a general, qualitative comparison of the rabbit and guinea pig sample populations. The test showed significant differences (P<0.001) between the general bacterial populations of rabbits and guinea pigs. A Mann-Whitney test [7] of the entire populations present in the rabbit and guinea pig faecal samples also showed significant differences (P<0.05) between bacterial species present in the two different animals.

Hence, although six different phyla could be identified, only two of them were present (Firmicutes and Bacteroidetes) in relatively large numbers in all animals, with Fibrobacteres also present at a high level in one of the guinea pigs.

### Comparison of faecal samples from domesticated rabbits and rectal samples from wild rabbits

**Fig 2:**
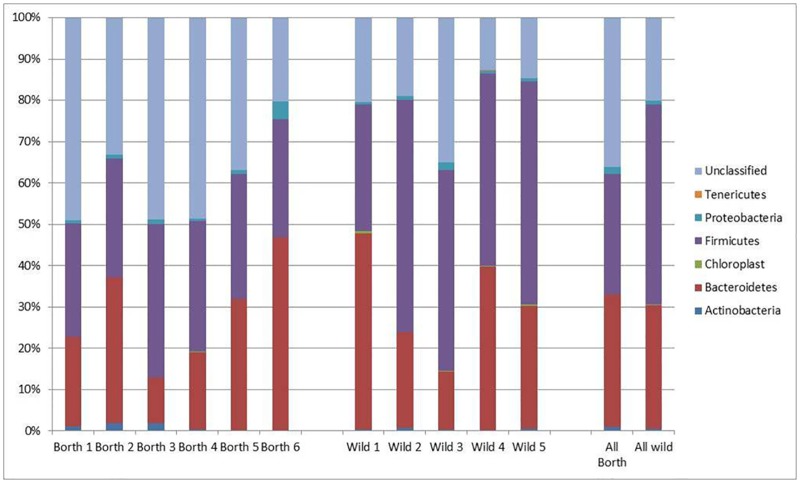
Percentage of each phylum present in fresh faecal samples collected from domesticated rabbits and rectal samples collected from wild rabbits together with the percentage of sequences which could not be classified within a particular phylum.

The range of phyla detected was very similar to that seen in the comparison between the domesticated rabbit and guinea pig faecal samples: Actinobacteria; Bacteroidetes; Firmicutes; Proteobacteria; and Tenericutes being detected. In addition there were a number of unclassified sequences. As seen with the rabbit faecal samples previously, there were no sequences from Fibrobacteres. Although five different phyla could be identified, and four of them were present in all animals, only two of them (Firmicutes and Bacteroidetes) were present in relatively large numbers in all animals. Fig 2 shows the relative abundance of each of these phyla within the animals.

The relative abundance of the Bacteroidetes was similar in both groups (32.3% and 29.9% in domesticated and wild rabbits respectively). Proteobacteria levels were also similar (around 1–2% in both groups) with the exception of a single domesticated animal where the level was 4.2%. Tenericutes sequences were absent from the domesticated animal but detected at a low level in two of the wild animals; a single copy in one animal, and around 0.1% of the total number in the other.

The major differences between the two groups were seen in terms of the proportions of Firmicutes and unclassified organisms. In the domesticated rabbits Firmicutes constituted 29.0% of the sequences, whilst unclassified sequences comprised 36.2%. In the wild rabbits these figures were 48.4% and 20.0% respectively.

In total there were 120 OTUs which were classified as being Bacteroidetes. Of these 22 were specific to the domesticated animals and 53 specific to the wild animals, with 45 common to both. Some of the sequences found in both groups of animals had up to 10 fold difference, but there were no cases where the differences were as high as those seen between those in the faecal samples from domesticated rabbits and guinea pigs, which reached 1000 fold differences in some cases.

In addition to there being more Firmicutes sequences in the wild rabbits, relative to the domesticated ones (48.4% versus 29.0%), there were also more Firmicutes OTUs in the samples from the wild animals; 166 OTUs were specific to the wild rabbits, with 27 specific to the domesticated animals and 212 found in all eleven rabbits investigated. As with the Bacteroidetes, the sequences found in both groups of rabbits tended to be present at similar levels, with most being present at similar levels.

There was a higher proportion of unclassified sequences in the samples collected from domesticated rabbits, relative to the wild ones (36.2% versus 20.0%). However the number of unclassified OTUs was lower with only 9 OTUs which were specific to the samples from domesticated animals relative to the wild ones (109 OTUs), with 175 OTUs common to rabbits from both groups. Within the unclassified OTUs present in both sets of animals there was evidence of some sequences which were present at approximately equal levels, whilst others were orders of magnitude (1000 fold and more) different. Thus, by inference, it would appear that some of bacterial species from which the unclassified sequences were isolated are having their proportional numbers regulated by the type of diet eaten by the rabbit.

Cochran’s Q test was used to provide a general, qualitative comparison of the domesticated and wild rabbit populations. The test showed significant differences (P<0.001) between the faecal/rectal bacterial populations of wild and domesticated rabbits. A Mann-Whitney test also showed significant differences (P<0.05) between bacterial species present in the two groups of animals.

### Comparison of samples from different organs of the digestive tract of wild rabbits

Given the difference in physiological differences between different organs, it might be expected that there would be differences in the microbial communities in the different organs. For example the pH of the stomach has a low pH (1.5 to 2.0) whilst the small intestine is higher (around 7.2) and then slightly lower again in the hindgut (around 6.0 to 6.5). Likewise the dry matter content varies throughout the tract [8].

**Fig 3:**
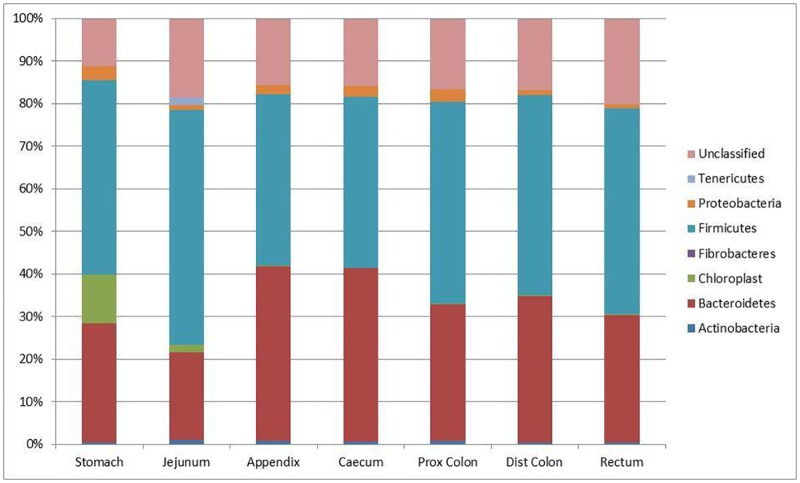
Percentage of each phylum present in samples collected from the stomach, jejunum, caecum, appendicular caecum, proximal colon, distal colon and rectum of wild rabbits together with the percentage of sequences which could not be classified within a particular phylum. Data presented are the mean values of 5 samples per region.

The phyla detected included those described above for other rabbit samples: Actinobacteria; Bacteroidetes; Firmicutes; Proteobacteria; and Tenericutes. In addition Fibrobacteres sequences were detected in samples from the stomachs of three of the rabbits, although always at <0.1% of the total population. Again there were a large number of unclassified sequences. In all seven organs investigated the major phyla detected were again Firmicutes and Bacteroidetes. The relative abundance of each of these phyla in the various organs is shown in Fig 3.

Actinobacteria were found throughout the digestive tract but in all organs the relative abundance was low, ranging from 1.0% (jejunum) to 0.4% (rectum). In total within the digestive tract there were 12 different OTUs detected, and with the exception of the stomach (where there were only 7 OTUs) all of them were detected in all areas of the tract.

Proteobacteria were found throughout the digestive tract, with the highest numbers in the stomach (3.5% of all sequences), with all other organs showing similar levels. In total 13 proteobacterial OTUs were observed with each organ containing 8 to 10 OTUs. Six of these OTUs were found in all organs. OTU0963 was an interesting sequence as it constituted about 2.5% of the total number of sequences identified in the stomach, but was only found at background levels (3 and 1 copies in the appendicular caecum and rectum respectively) in other organs.

A single Tenericutes OTU (OTU0462) was found in five of the organs (jejunum, appendicular caecum, caecum, distal colon, and rectum). With the exception of the jejunum where it constituted 1.8% of the total sequences it never constituted more than at trace levels (e.g. 0.03% in the rectum) and was only seen in two of the wild rabbits. By performing a BLAST search, OTU0462 was identified as having 96% identity to a member of the genus *Mycoplasma*.

Members of the Bacteroidetes phylum were found throughout the entire digestive tract and constituted 20–30% of the total number of sequences detected in the stomach, jejunum, proximal colon, distal colon and rectum, where they were the second most abundant phylum. In the case of the caecum and appendicular caecum, the Bacteroidetes sequences constituted around 40% of the total number of sequences, which was approximately the same number as those identified as Firmicutes in these two organs. In total 150 Bacteroidetes OTUs were identified with numbers of OTUs varying between organs (38 in the proximal colon to 113 in the stomach). In addition the OTUs present in any particular organ had some degree of inter-organ variation, although this tended to be for the less abundant Bacteroidetes OTUs, with a number of the more common ones being found at relatively high abundance throughout the entire tract.

The remaining identifiable phylum, the Firmicutes, was the most abundant in five of the organs: stomach; jejunum; proximal colon; distal colon; and rectum. In these five organs the actual values ranged from 45.5% in the stomach to 55.2% in the jejunum. In the remaining two organs (the caecum and appendicular caecum) the value was 40.3%, which was approximately the same as abundance as the Bacteroidetes. In total 421 Firmicutes OTUs were detected in the samples from wild rabbits. The lowest number was seen in the stomach which had 245 OTUs. All other organs ranged from 313 (jejunum) to 365 (appendicular colon).

There were also 314 OTUs which could not be classified. The number of unclassified OTUs per organ varied from 154 in the stomach to 284 in the rectum. and some of these were found in all organs. It is worth noting that many of these OTUs were found at relatively low levels, but a few examples existed (e.g. OTU0067) at around 1% of the total number of sequences present in all of the different organs.

Cochran’s Q test showed significant differences (P<0.001) between the bacterial populations in the different organs of these wild rabbits.

## Discussion

It has already been reported that different species have different microbial communities in their respective digestive tracts. However this is the first time that samples from the guinea pig have been investigated, and also the first time that a comparison has been made between samples collected from two different species of caecotrophagic animals. While the animals were co-housed, and given access to the same food, the actual intake by each individual animal was not monitored. It is anticipated that differences between species probably had some dietary bias. However, allowing animals to select their own choice of diet is probably a more accurate reflection of the pattern likely to be seen in the two species, rather than drawing conclusions from a diet which may not accurately reflect those normally given to these animals.

The first report of the composition of the microbial community of the digestive tract of the rabbit took place around a decade ago [6] and, based on a relatively small number of sequences, concluded that the microbial population was unlike those described for other herbivores. These results were corroborated by a further publication using a larger dataset [9]. In the first of these papers no members of the Bacteroidetes group was reported, and in the latter the Bacteroidetes represented only 4% of the sequences. These values were similar to those seen in [5] for samples from the equine hindgut, but much lower than those seen in samples from the rumen e.g. [10] where Bacteroidetes numbers were around 35% of the population. In both cases the major group of organisms identified in the rabbit showed most similarity to the Firmicutes.

More recently next generation sequencing (NGS) has allowed a more extensive investigation of the community structure to be undertaken and the first time this technology was applied to the digestive tract of the rabbit resulted in the majority (range 83.3 to 99.7%) of the identifiable sequences classified as either Bacteroides or Firmicutes [11]. Further use of NGS has demonstrated that these two groups of organisms dominate the caecal population from an early stage in the animal’s life, although there is a shift in composition over time, with Bacteroidetes at roughly double the abundance of Firmicutes in early life (14 days) but gradually Firmicutes became more abundant until they comprised over 90% of the population by 80 days [12]. The current data also show that over 90% of the identifiable sequences are either Bacteroides or Firmicutes; 96% for the domesticated rabbits and 98% for guinea pigs. However unlike the observations of [12] where sequences from Firmicutes, were the most abundant group in adult animals, the rabbits here show a relatively even split (51% Bacteroidetes and 45% Firmicutes) and the guinea pigs show that the majority of identifiable sequences are from Bacteroidetes (86%). It is worth noting that this is the first time that the DNA being analysed was extracted from a microbial community present in rabbits which had not been fed on a pelleted diet, instead having access to vegetable trimmings, hay; fresh fruit and wild leaves. Therefore we postulate that higher Bacteroidetes numbers seen here may be associated with higher fibre content in the diet.

In the case of the wild animals, which were assumed to have existed on a diet based primarily of leaves and grasses (in keeping with the chloroplast sequences detected in the digestive tract) the percentage of sequences which were Bacteroidetes was around 40% in the caecum and appendicular caecum, and 20–30% in all other organs investigated. This supports the earlier observation with domesticated rabbits where we found high levels of Bacteroidetes. Again this may be a reflection of the higher fresh fibre content of the diet, although it is interesting to note that the samples from wild animals, which would presumably contain higher levels of fresh forage than given to the domesticated rabbits, had less Bacteroidetes sequences. Suggestions of dietary preferences in rabbits are in keeping with previous speculation [13] where wild rabbits were found to have a higher preference for concentrates when available, rather than fibrous feeds, although feed available in the wild in the current work (Wales) is likely to have more moisture associated with it than this previous work (Spain). However, as with the other observations, for all organs the sum of Firmicutes and Bacteroidetes constituted 95% or more of the sequences which could be identified and assigned to a phylum.

Members of the Bacteroidetes phylum were found throughout the entire digestive tract and constituted 20–30% of the total number of sequences detected in the stomach, jejunum, proximal colon, distal colon and rectum. In these organs Bacteroidetes was the second most abundant phylum. In the case of the caecum and appendicular caecum, the Bacteroidetes sequences constituted around 40% of the total number of sequences, which was approximately the same number as those identified as Firmicutes in these two organs. In total 150 Bacteroidetes OTUs were identified with numbers of OTUs varying between organs (38 in the proximal colon to 113 in the stomach). In addition the OTUs present in any particular organ had some degree of inter-organ variation, although this tended to be for the less abundant Bacteroidetes OTUs, with a number of the more common ones being found at relatively high abundance throughout the entire tract.

The remaining identifiable phylum, the Firmicutes, was the most abundant in five of the organs: stomach; jejunum; proximal colon; distal colon; and rectum. In these five organs the actual values ranged from 45.5% in the stomach to 55.2% in the jejunum. In the remaining two organs (the caecum and appendicular caecum) the value was 40.3%, which was approximately the same as abundance as the Bacteroidetes. In total 421 Firmicutes OTUs were detected in the samples from wild rabbits. The lowest number was seen in the stomach which had 245 OTUs. All other organs ranged from 313 (jejunum) to 365 (appendicular colon).

As mentioned above, there were also 314 OTUs which could not be classified. Clearly the role of the organisms corresponding to these sequences is unknown. However examples such as sequence OTU0067 which appears throughout the digestive tract, at around 1% of the microbial community is likely to have an important role to play within the animal.

If the current observation regarding the increased levels of Bacteroidetes is associated with differences in fibre content in the diet, it is interesting to note that this was not the pattern seen in studies of rumen content of animals fed on either forage, high grain, or mixed diets, where the Firmicutes and Bacteroidetes remained relatively constant across all three diets [14]. However, it is in keeping with studies on children in rural Africa who were shown to have have a higher abundance of Bacteroidetes than the equivalent age group in Europe e.g. [15] most probably due to a higher fibre content in their diet.

Both of the major phyla (Bacteroidetes and Firmicutes) and also the unclassified group are extensively represented in all of the samples investigated, irrespective of species and also of organ. However, within each of these phyla there are a number of OTUs which are specifically found in either the rabbit or the guinea pig. Moreover, even in OTUs which were found in samples from both species there were examples of a clear bias in their relative abundance, with the number of copies of a particular sequence typically being 100 to 1000 times more abundant in the apparently preferred species. Therefore this argues that there is a high level of species-specificity in terms of the population of microbes found within each of these two caecotrophagic species, albeit that the bacteria show some degree of relationship.

It is also worth noting that the rabbit samples, irrespective of domestication level lacked any Fibrobacteres other than a baseline level in the stomach of three of the wild rabbit. This is an observation in keeping with previous reports. For example in [6] the highest similarity to any member of the *Fibrobacter* genus was at 85.7%, suggesting that this organism was not a member of the Fibrobacteres phylum. In [9], again no member of this genus was found, although the authors speculate that this may be due to the re-enforcement of the hypothesis of [16] where it was suggested that the 16S *rRNA* gene of *F*. *succinogenes* may be prone to less efficient amplification by PCR, relative to other species. However even with the use of NGS [12] members of the *Fibrobacter* genus were discounted as being numerically important. This is in contrast to the rumen environment where, depending on the conditions, members of the *Fibrobacter* genus can be relatively abundant [17] or in the caecum of the horse [18]. However the situation in the samples from the guinea pigs is less clear, with five of the samples showing the Fibrobacteres to be either absent or at baseline detection (i.e. single copies of the sequence per sample), whilst the final sample has this single Fibrobacteres OTU present at 10.9% of the total sequences. This suggests that the Fibrobacteres are of little importance to digestion in the rabbit, but in at least one of the guinea pigs studied it is an important organism.

Thus it appears that there are major differences in the microbial population of the digestive tracts of the rabbit and the guinea pig, and between organs within a single animal. While some of this may be due to dietary preferences between the animals, it nevertheless suggests that it is unlikely that there is a single inter-species microbial population which is responsible for allowing an animal to live a caecotrophagic lifestyle.

## Materials and methods

### Sample collection from domesticated rabbits and guinea pigs

Ten adult rabbits and ten adult guinea pigs were co-housed in an indoor run at Borth Animalarium in West Wales. All animals had been given access to the same diet for several months and were given *ad libitum* access to the same range of food: rabbit muesli (Badminton Albion Bunny Munch Ultra); guinea pig nuggets (Burgess Excel Tasty Nuggets Guinea Pig Food); a mixture of vegetable trimmings; willow bark; hay; straw; a mixture of fresh fruit; and wild leaves. In addition they had constant access to fresh water. Animals tended to eat the respective species-specific muesli or nugget, and guinea pigs were more likely to eat vegetable trimmings, fresh fruit and leaves, with rabbits eating more hay and bark, although there was a degree of shared eating patterns.

Fresh faecal samples were collected from the first six animals from each species which were seen defecating. In all cases samples collected were hard faececs (i.e. not caecotrophs). Samples were stored in individual thermos flasks and transported back to the lab for DNA isolation.

### Sample collection from wild rabbits

Fresh digestive tracts were collected from local wild rabbits which had been shot for food. The tracts were collected and transported on ice to the lab within 30 minutes of the animal being killed. Digesta was removed from the stomach, jejunum, caecum, appendicular caecum, proximal colon, distal colon and rectum. DNA was extracted from the digesta as described above.

### DNA extraction

On returning to the lab samples were frozen and stored -20°C, after which DNA extraction was carried out using a QIAamp^®^ DNA Mini Stool Kit (Qiagen Ltd.; West Sussex, England) following the manufacturer’s standard protocol with the exception of increasing the initial incubation from 70°C to 95°C for 5 min, which the manufacturers suggest helps with the lysis of Gram-positive bacteria which are known to be present in large numbers in the digestive tract of the rabbit e.g. [6].

Once DNA was extracted the purity and concentration of the DNA was assessed using a BioTek Epoch Spectrophotometer System using measurements at A_260_ and A_280_.

### PCR conditions

PCR was performed to amplify part of the V2-V3 region 16S *rRNA* gene of each sample using the reverse primer 355R (5’-CTG CTG CCT CCC GTA GGA GT-3’) in all reactions [19] and the basal forward primer 27F (5’-CCA TCT CAT CCC TGC GTG TCT CCG ACT CAG-3’) linked to a reaction sample-specific “bar code” 10mer at the 5’ of the primer. Approximately 1ng of DNA was used per reaction in a reaction cocktail containing both primers (100nM each), 200μM of each dNTP, the manufacturer’s buffer (supplemented to 1.8mM MgCl_2_) and 1.25U FastStart high fidelity enzyme (Bioline) in a final reaction volume of 25μl. Amplification was performed with the following steps: 95°C hot start for 2 min; 21 cycles (94°C for 30 sec, 52°C for 30 sec, 64°C for 30 sec); and a final extension at 64°C for 7 min. The presence of amplicons was verified at the end of the PCR stage by electrophoresis.

### Preparation for sequencing

The purity and concentration of the amplicons were assessed using a BioTek Epoch Spectrophotometer System. Two clean-up procedures (AMPure XP bead clean-up and E-Gel agarose gel electrophoresis) were carried out to remove undesired, short fragments. Sample concentrations were then normalised (120ng/μl) and equal quantities of all samples pooled for all further stages. The success of the two clean-up procedures in removing unwanted DNA fragments was assessed using an Agilent 2100 Bioanalyzer (Agilent Technologies, Santa Clara, CA, USA), following the protocol detailed in the Agilent High Sensitivity DNA Kit Quick Start Guide (Agilent Technologies, 2009).

The sample library underwent emulsion PCR (emPCR) as the final step in preparation for Ion Torrent sequencing. The library’s fragments were attached to Ion PGM Template OT2 400 Ion Sphere Particles (Life Technologies, Carlsbad, CA, USA) and then clonally amplified. The protocol described in the Ion PGM Template OT2 400 Kit User Guide (Life Technologies) was followed exactly. The Ion OneTouch 2 Instrument (Life Technologies) was prepared as per the User Guide’s instructions.

### DNA sequencing

The Ion Torrent sequencing procedure was run on an Ion Torrent PGM System (Life Technologies, Carlsbas, CA, USA) using an Ion PGM Sequencing 400 Kit, following the manufacturer’s protocol, as described in the Ion PGM Sequencing 400 Kit manual (Life Technologies).

### Analysis of sequence data

Initial quality filtering of DNA sequence reads was performed with standard settings by the Ion Torrent PGM platform. Final library output files were then filtered by minimum read length (250 base pairs) to remove any remaining short reads, and individually barcoded sample files were then merged for OTU clustering (≥97% identity), performed through the CD-HIT-OTU program [20]. Sequences were checked to avoid the occurrence of identifiable chimera at this time. Unequal numbers of sequence reads per sample were obtained from the sequencing process, necessitating normalization of the dataset for statistical analyses. This was performed using the script daisychopper.pl (http://www.genomics.ceh.ac.uk/GeneSwytch/Tools.html), which randomly resampled the dataset to ensure that all samples contained equal numbers of reads, matched to the lowest number of reads obtained for an individual sample, thus allowing samples with unequal sequence coverage to be statistically compared [21], [22].

The Ion Torrent PGM classified the DNA sequences from the sample library into distinct reads or OTUs. The PGM output also included data on the relative abundance of each OTU in each sample. An initial identification of OTUs was performed using the Ribosomal Database Project (RDP) Bayesian classifier [23], [24]. This identification relied on taxonomic differentiation based on identity levels of ≥97% being defined as the same OTU [25] and classification at the level of phylum being ≥90% identity.

Qualitative differences (present/absent binary scores) between different regions of the tract were compared using a Cochran’s Q test [26].

Individual sequences which were of particular interest were identified using Basic Local Alignment Search Tool (BLAST) analysis [27] to both phylum, and where possible genus, level. Percentage abundance calculations were performed for classification at phylum level and expressed in bar graph format.

## Supporting information

S1 File Supplementary data. (DOCX)
