# Supplementary material for: Comparison of the microbial population in rabbits and guinea pigs by next generation sequencing
Source: PLoS One. 2017 Feb 9;12(2):e0165779. doi: 10.1371/journal.pone.0165779 (PMC5300138; doi:10.1371/journal.pone.0165779)
Supplement: S1 File — (DOCX) [file pone.0165779.s001.docx]

**Rabbit Guinea Pig Caecum Caecum Proximal Distal**

**Faeces Faeces Stomach Jejunum Appendix Main Colon Colon Rectum**

**Phylum Class n=6 n=6 n=5 n=5 n=5 n=5 n=5 n=5 n=5**

Actinobacteria Actinobacteria 1450 1017 26 300 1567 1975 2067 1396 944

Bacteroidetes Bacteroidetes 2 422 0 0 1 0 0 0 1

Bacteroidetes Bacteroidia 53474 167771 1146 4634 71754 104453 70292 92170 55662

Bacteroidetes Flavobacteria 10 15130 59 37 104 510 109 413 250

Bacteroidetes Sphingobacteria 7948 22010 67 14 7 4 0 1 26

Fibrobacteres Fibrobacteria 0 2238 5 0 0 0 0 0 0

Firmicutes Bacilli 128 0 1 71 337 528 464 498 436

Firmicutes Clostridia 89299 39920 2228 14525 88978 131760 127484 155035 113174

Firmicutes Erysipelotrichia 4825 7059 47 75 1264 1938 1520 2095 1579

Firmicutes Negativicutes 213 636 1 202 12 19 26 75 74

Proteobacteria Alphaproteobacteria 7172 12992 31 253 1869 2478 2884 3645 4133

Proteobacteria Betaproteobacteria 684 387 19 96 931 1798 1710 1333 641

Proteobacteria Deltaproteobacteria 293 150 3 46 635 1293 1202 830 456

Proteobacteria Epsilonproteobacteria 376 548 17 122 1963 3354 3566 1239 958

Proteobacteria Gammaproteobacteria 1835 1184 119 638 428 508 564 847 710

Proteobacteria Zetaproteobacteria 0 138 1 0 0 0 0 0 0

Spirochaetes Spirochaetes 3 4891 14 0 0 2 0 0 8

Tenericutes Mollicutes 944 2 4 442 240 370 267 352 308

**Table A**

Total OTUs per class detected with non-standardised data. Only samples where the identity value was 90% or more have been included.
